# A feasibility study on two tailored interventions to improve adherence in adults with haemophilia

**DOI:** 10.1186/s40814-020-00723-w

**Published:** 2020-12-01

**Authors:** J. W. Hoefnagels, K. Fischer, R. A. T. Bos, M. H. E. Driessens, S. L. A. Meijer, R. E. G. Schutgens, L. H. Schrijvers

**Affiliations:** 1 Division Internal Medicine and Dermatology, Van Creveldkliniek, University Medical Center Utrecht, PO Box 85500, 3508 GA Utrecht, The Netherlands; 2 Netherlands Haemophilia Patient Society (NVHP), Nijkerk, The Netherlands; 3 Utrecht University of Applied Sciences, Utrecht, The Netherlands

**Keywords:** Lifelong treatment, Illness acceptance, Adherence, Acceptance and commitment therapy, Self-management, Congenital disease

## Abstract

**Introduction:**

Haemophilia is a congenital bleeding disorder mainly affecting males. To prevent bleeding, patients need to perform regular intravenous injections (prophylaxis) throughout life. Non-adherence often occurs. Problems with acceptance or self-management appear to be the main reasons for non-adherence in haemophilia. The aim of this study was to test the feasibility and effects of two interventions focussed on acceptance (face-to-face) and self-management (online).

**Methods:**

Patients with severe haemophilia and acceptance or self-management problems were eligible. The face-to-face group intervention was based on Acceptance and Commitment Therapy (ACT) (8 sessions/6 months, target *N* = 8 participants). The online intervention was based on a successful online programme in rheumatoid arthritis (5–8 modules/2 months, target *N* = 8). Both interventions were designed according to the MRC framework in collaboration with the patient society and experts. We compared adherence (VERITAS-Pro, optimum 0), quality of life (SF-36, optimum 100) and illness perception (BIPQ, optimum 0) before start (T0) and after 2 months (T2). Feasibility criteria were as follows: completion of training by > 50% of participants and ability to collect at least 80% of outcome parameters.

**Results:**

The face-to-face intervention was feasible (89% enrolment and recruitment, 100% retention). One hundred percent of the outcome parameters was collected. Results were promising: although adherence (VERITAS-Pro) was stable (from 64 to 62 points), quality of life (SF-36) showed a clinically relevant improvement (> 5 points) in five of eight domains. Illness perception (BIPQ) showed a clinically relevant increase from 47 to 39 points. Patient evaluation was positive.

The online intervention, however, was infeasible: enrolment was only 20% (6/30). Only three patients signed informed consent (recruitment 10%), and none completed more than one module (retention 0%). Consequently, the online intervention was terminated.

**Conclusion:**

The face-to-face acceptance intervention was considered feasible with promising results. Unfortunately, the online intervention was infeasible and therefore terminated. These findings suggest that adapting effective interventions to other settings does not guarantee success, despite the use of established methodology and patient participation. Population differences (only male participants, congenital disease) could be an explanation for failure of the online intervention in haemophilia despite success in rheumatoid arthritis.

**Trial registration:**

NL55883.041.16

## Key messages regarding feasibility

- What uncertainties existed regarding the feasibility?
  Uncertainty regarding recruitment of patients struggling with illness acceptance and self-management. Uncertainty regarding the ability of patients to complete the programmes.
- What are the key feasibility findings?
  The face-to-face group intervention regarding illness acceptance was feasible and showed promising results on quality of life and illness acceptance with stable adherence. The online intervention was infeasible due to recruitment problems.
- What are the implications of the feasibility findings for the design of the main study?
  The face-to-face intervention will be evaluated in a larger group. The online training was removed from the study.

## Background

The introduction of intravenous clotting factor replacement therapy has enabled the substitution of the missing clotting factor in haemophilia [1]. This therapy has been administered to treat bleeds (on demand) or as regular replacement therapy (prophylaxis) to prevent bleeds [2]. This intravenous prophylactic treatment is self-administered by the patient at home, approximately 3 to 3.5 times per week [1]. For effective prevention of bleeding, high adherence to this prophylactic treatment is crucial. To maintain minimum clotting factor levels and preserve joint health, prophylaxis should be continued lifelong without interruption [1].

Non-adherence to prophylaxis (i.e. ≥ 25% missed infusions, or ≥ 25% dose change and/or 30% deviation in timing [3]) occurs in approximately 50% of Dutch adults with severe haemophilia [4–7]. Non-adherence and inadequate treatment of bleeds can cause irreversible damage, especially in a joint or the central nervous system [8]. Patients who stopped or interrupted the treatment had a significantly worse joint status than patients who did not stop (HJHS: 23 vs 14 points *P* = < 0.01 and Pettersson score: 16 vs 5 points *P* = < 0.01) [9]. This joint damage eventually results in a lower quality of life and reduced labour force participation [10, 11], which stresses the importance of high adherence levels.

In a previous qualitative study, illness acceptance problems and lack of self-management skills were identified as important reasons for non-adherence [12]. Patients with acceptance problems mostly stopped prophylaxis or used prophylaxis intermittently (e.g. only on demand or skipping doses). In case of self-management problems, patients failed to administer prophylaxis due to inadequate routine, forgetfulness and the complexity of the self-management skills required [12]. Our hypothesis was that both groups could benefit from a tailored intervention to improve adherence.

Therefore, two tailored interventions were developed. The first intervention was focussed on improvement of illness acceptance using a haemophilia-adapted version of Acceptance and Commitment Therapy (ACT). The second intervention was focussed on improvement of self-management through an online programme including peer support. The aim of this study was to test the feasibility and effectiveness of both interventions (1) acceptance programme (face-to-face) and (2) self-management programme (online) in patients with severe haemophilia using prophylaxis.

## Methods

**Fig. 1:**
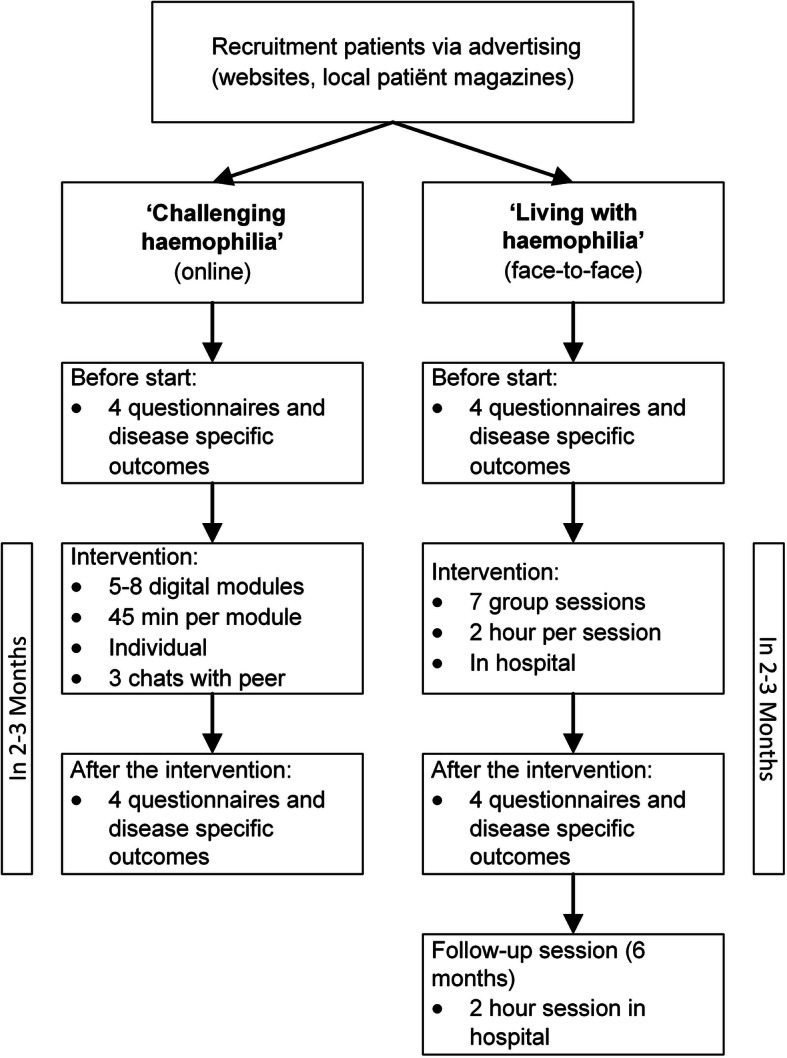
Study design

The study design of this feasibility study [13] is shown in Fig. 1. Patients who experience difficulties with accepting haemophilia were identified by the haemophilia treatment team and invited to join face-to-face group training: ‘Living with haemophilia’. This group training comprised of seven sessions and one follow-up session, guided by a trained haemophilia caregiver (social worker and nurse). Patients who experienced difficulties with self-management skills could join an individual online training: ‘Challenging your haemophilia’. This online programme included 5–8 modules, guided by a trained peer. We used the CONSORT 2010 guidelines for transparent reporting studies [14]. The trial registration is NL55883.041.16

### Face-to-face group training focussed on acceptance: ‘Living with haemophilia’

The face-to-face group training ‘Living with haemophilia’ was focused on improving illness acceptance, which could lead to a higher adherence to prophylaxis. This face-to-face group training was based on the Acceptance and Commitment Therapy (ACT) approach [15]. ACT is an evidence-based intervention using cognitive behaviour strategies and mindfulness, creating distancing from negative thoughts and words, emotions or physical sensations based on relational frame theory [15, 16]. This training addressed topics like creating awareness about thoughts, discussing self-realization and exploring personal values in life (see Table 1 for more details). Group dynamics and peer contact are an important aspect of the training [17]. ACT has been successfully used in patients with a chronic disease, e.g. diabetes, HIV and chronic pain [16–19]. The original ACT training was adapted and modified to the haemophilia population, in collaboration with the department of Psychology (University Utrecht) and support of the Netherlands Haemophilia Patient Society. Based on the ACT principles and previous studies, the training consisted of seven evening-sessions of 2 h per session and one follow-up session after 6 months [20, 21]. The training was fully scripted in a handbook with specific exercises and metaphors. All sessions were supervised by two ACT-qualified haemophilia healthcare professionals. Because of logistical reasons, dinner was provided at the start of each session.

**Table 1:** Overview of both trainings

	Session/module	Aim of the session
‘Living with haemophilia’ (face-to-face)	1.Control	Creating realization in how a patient is controlling thoughts, feelings and physical sensations. Discussing if this way of handling thoughts, feelings and physical sensations is effective or not.
	2. Acceptance	Creating space for tiresome thoughts, feelings and physical sensations. Acceptance is part of tolerating challenging experiences patients cannot get rid of.
	3. Letting go of thoughts	Creating realization about thoughts and how thoughts arise. Discuss if thoughts always are effective and if not how to transform them by changing them.
	4. Self	Distancing ourselves from strict or rigid beliefs. Discuss who someone is and someone’s identity. Discussing if this vision about yourself is real or if they are just thoughts, feelings and physical sensations. Discuss who someone wants to be.
	5. Values	Creating realization in what a patient must do, can do and wants to do. Discussing which values are important for the patient and what he really wants in life.
	6. Handling	Discussing barriers, motivators and strategies for things patients really want to do. Creating realization of someone’s short and long time reward. Developing concrete plans.
	7 Looking back and forward	Summarizing on the six concepts (control, acceptance, letting go thoughts, self, values and handling) and discussing used metaphors and exercises.
	8. Follow-up session	Discussing progress and providing additional advice.
‘Challenging your haemophilia’ (online)	1. Willing	Which values are important, learning about self-management and setting personal goals
	2. Knowledge	What is haemophilia, advantage and disadvantages of haemophilia and how to intergrade prophylaxis regimen into daily life
	3. Being able	Infusing tips, taking and holding your own control, making responsible choices, tackling problems, communication and giving and receiving feedback
	4. Living together	Communication about haemophilia with others, haemophilia and its impact on relationships and sexuality and impact of haemophilia on children
	5. Exercise and sport	Differences in goals and levels of exercising, making consensus choices about exercising, current exercise habits, impact of exercise on daily life and importance of taking a day off
	6. Work	The combination of work and haemophilia, potential obstacles and how to tackle them
	7. Looking ahead	Evaluating and setting personal goals

### Online training focussed on self-management: ‘Challenging your haemophilia’

The online training ‘Challenging your haemophilia’ was aimed at improving self-management, which could lead to a higher adherence to prophylaxis. This online training was based on a successful comparable online training developed to improve self-management in rheumatic arthritis [22] which was based on the Arthritis Self-Management intervention (ASMP) of Stanford University [23]. This training included 5 mandatory and 3 additional modules available on a secured website. This modules including exercises, short videos, written information and the possibility to chat with trained (*N* = 5) peer trainers. The peer trainers received a formal peer trainers’ training from an external specialized agency. Each module took approximately 45 min; training had to be completed within 2 months. Details of the modules are shown in Table 1. The modules are adapted from the rheumatic arthritis format towards haemophilia, following the six steps of the Medical Research Council (MRC) framework [24]. First, the evidence base was identified by a review of the literature [25], followed by a problem analysis (theory development) [12] and a modelling process based on existing material [22]. Subsequently, a prototype was developed in collaboration with some patients and a delegation of the patient organisation (three panel sessions), followed by an evaluation of the look and feel of this prototype during a patient panel meeting followed by field usability testing [24].

### Participants

For both interventions, male adults with haemophilia who were prescribed prophylactic treatment were eligible. Ability to understand written language and speak Dutch was a prerequisite. Patients diagnosed with a serious psychiatric disorder interfering with the training were not eligible. For the online training, access to internet was a prerequisite. Based on the need to follow the conversations at the online platform and to perform an oral evaluation with each participant, the maximum number of participants per group was set at eight. At the beginning of each week, all patients visiting the clinic were discussed (multidisciplinary) and eligibility was considered. Patients who were eligible were informed about this study (and both interventions) by their health care provider or through various digital platforms (different websites, newsletters of the patient society, social media). Patients received information about both interventions and could choose between both trainings based on their personal preference and their opinion regarding the reason for non-adherence: struggle with acceptance of haemophilia or with self-management. Informed consent was signed prior to the study.

### Data collection

For both interventions, data was collected before start and directly after the intervention. The primary outcome of both interventions was adherence and secondary outcome was quality of life. Additionally, each intervention included an intervention-specific outcome measurement: participants in the face-to-face intervention completed an illness perception questionnaire and those in the online intervention completed a health education impact questionnaire.

Adherence was measured by assessment of three domains (skipping, dose changes, time changes) based on a pre-specified definition [3] and the Validated Hemophilia Regimen Treatment Adherence Scale –Prophylaxis [26] (VERITAS-Pro) questionnaire, with a range from 100 to 0 and optimum score of 0. Quality of life was measured using the Short Form-36 Health Survey Questionnaire (SF-36), with a range from 0 to 100 with an optimum score of 100 [27]. Intervention-specific secondary outcomes were illness acceptance and self-management. Acceptance was assessed by the Brief Illness Perception Questionnaire (Brief IPQ), with a range from 80 to 0 with an optimum score of 0 [28], and the Health Education Impact Questionnaire (HEIQ), with a range from 0 to 5 with an optimum score of 5 [29]. In the study protocol, the minimal important difference (MID) in outcome was pre-defined. For the SF-36, the MID is established on a 5-point increase [30]. The study team has decided to use the same MID for the other questionnaires. Questionnaire details including domains, scores ranges and MID are provided in Table 2. In addition, demographic variables (age, haemophilia severity, prescribed treatment and employment) were collected before start of the intervention from patients’ medical records. During the last session of the face-to-face group training, patient evaluation was performed using a short focus-group interview (10 min). The online training was evaluated using audio-taped individual phone interviews (10 min).

**Table 2:** Questionnaires used as outcome parameters

	Outcome	Questionnaire	Specifications	Minimal important difference
Used in both interventions	Adherence	VERITAS-Pro [26]	- 6 domains (Time, Dose, Plan, Remember, Skip and Communicate) - 24 multiple choice questions - Cumulative normalized total score ranging 0–100	Not official defined. We considered an increase of 5 point clinical relevant
	Quality of life	SF-36 [27]	- 8 domains (physical functioning (PH); role-physical (RH), Bodily Pain (BP), General health (GH), Vitally (V), Social functioning (SF), Role-emotional (RE) and mental health (MH). - 36 multiple choice questions	Increase by 5 points [30]
Face-to-face training	Illness perception	BIPQ [28]	- Only a total score, no domains - 8 multiple choice questions - Cumulative score ranging from 0 to 80	Not official defined. We considered an increase of 5-point clinical relevant

### Data analysis

We considered an intervention feasible if more than 50% of the patients completed the training and if more than 80% of the data for outcome parameters could be adequately collected. If not, the study team will consider early determination of adaptation.

Additionally, according to the definitions proposed by Craig et al., feasibility was expressed by a comparison of the number of patients on prophylaxis having problems with acceptance or self-management (eligible) with the number of patients willing to participate and signed informed consent (enrolment and recruitment), number of patients that followed and completed the training (retention) and time spent on the training practicability [24]. Patient characteristics were analysed using descriptive statistics. Data were analysed using descriptive statistics and if possible a Wilcoxon test (SPSS version 21). Patient evaluations were transcribed, summarized and thematic analysed. Themes were discussed by the research team.

## Results

This study was performed at the Van Creveldkliniek of the University Medical Center, Utrecht, the Netherlands. This clinic was established in 1964 and has always provided multidisciplinary treatment including designated and specialized physicians, nurses, physiotherapists and a social worker. The Van Creveldkliniek treats 250 adults with severe haemophilia, of which ± 50% has adherence problems, resulting in ± 125 eligible patients overall. For this feasibility study, our aim was to include *N* = 8 patients in each intervention.

### Face-to-face group training focussed on acceptance: ‘Living with haemophilia’

#### Recruitment

**Fig. 2:**
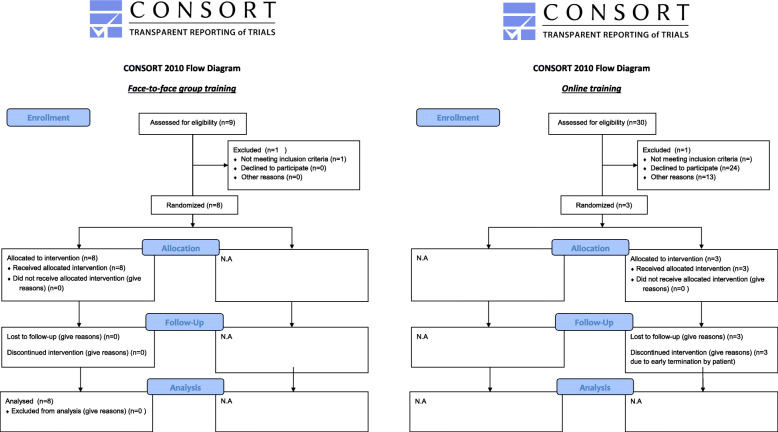
CONSORT flow diagram. This flow diagram template was downloaded from the official CONSORT website [14]

Figure 2 shows the CONSORT flow diagram, the left side concerns the face-to-face group training. Over a period of 2 months, nine patients were informed and invited to participate in the training, all were enthusiastic to participate in the training. All patients were screened before the start of the intervention. One patient did not start, because he was receiving individual psychological treatment. All included patients (*N* = 8) completed all seven training sessions. Consequently, enrolment and recruitment was 88% (8/9), and retention was 100% (8/8).

#### Feasibility

On two occasions, a participant could not attend a session because of work or illness. In these cases, the continuity of the training was covered by seeing the participant individually before the start of the next session. The eventual participation retention for this intervention was 100%. All participants joined the free dinner at the start of each session. The trainers evaluated the training as practical and achievable, and all outcome parameters were collected. The trainer’s preparation time before every session took approximately 15–30 min. This was considered achievable in a daily healthcare setting.

#### Participant characteristics

Patient and treatment characteristics are shown in Table 3. All participants had severe haemophilia A and had a median age of 38 years (range 27–51 years). The median prescribed frequency of intravenous clotting factor use was 3.2 infusions a week (range 0–7). One patient refused to take prophylactic treatment (but it was indicated), resulting in an infusion frequency of 0.

**Table 3:** Participant characteristics

	Adherence programme (*N* = 8)	Self-management programme (*N* = 3)
**Participant characteristics**	Number (%) or median (range)	
Severe haemophilia A	8 (100%)	3 (100%)
Age (years)	38.8 (27–51)	24 (20–32)
Prescribed frequency of prophylaxis infusions per week	3.2 (0–7)	3 (2–3)
Employment -Full time paid	6 (75%)	1 (33%)
**Adherence** (VERITAS-Pro, 100–0, optimum 0)		
Adherence	64	56
**Quality of life** (SF-36, 0–100, optimum 100)		
Physical function	60	95
Role-physical	13	100
Bodily Pain	57	62
General health	57	82
Vitality	63	65
Social functioning	63	75
Role-emotional	17	100

#### Baseline and follow-up assessments

**Fig. 3:**
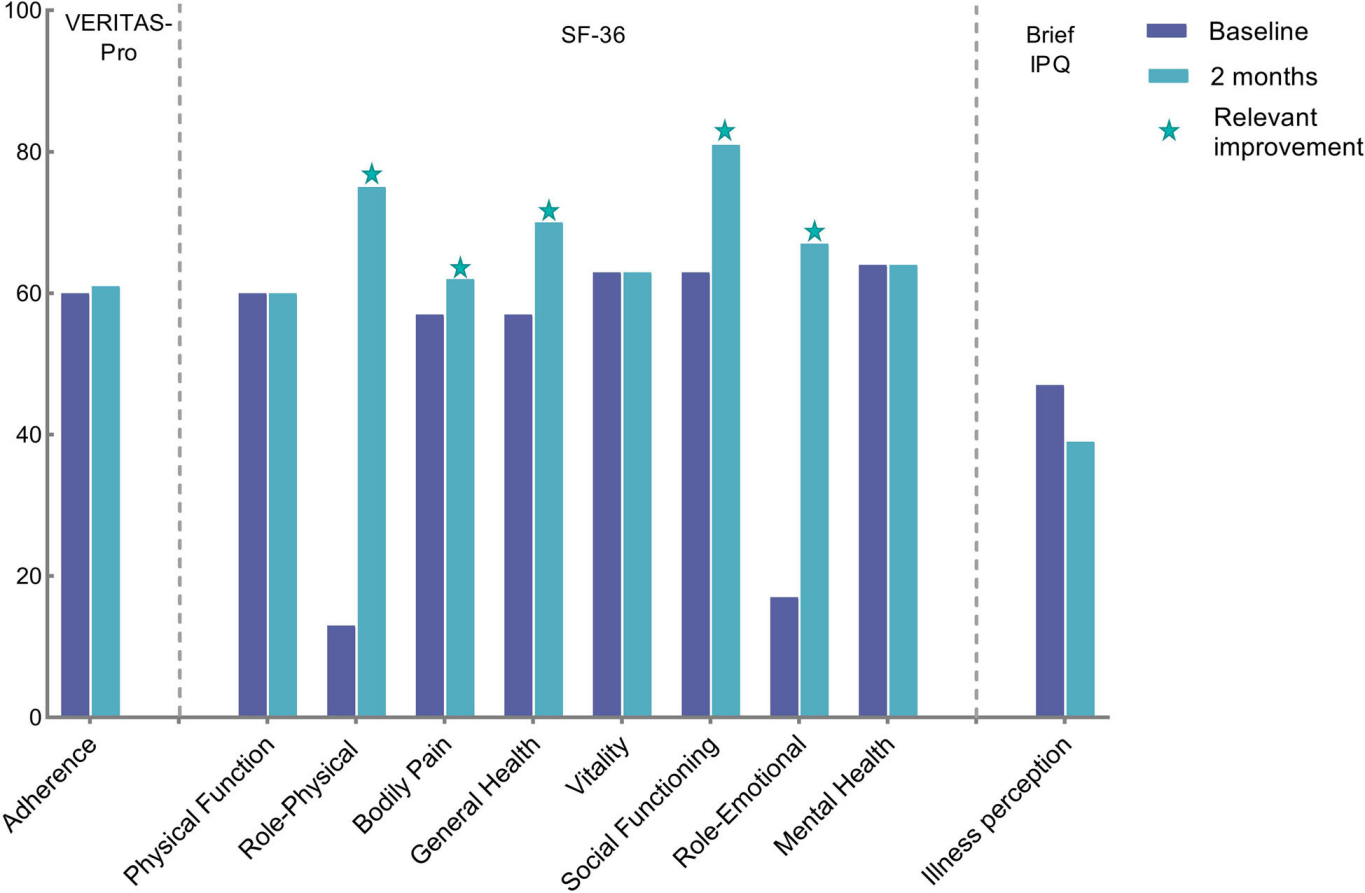
Baseline and follow-up assessments of the VERITAS-Pro, SF-36 and brief IPQ

Baseline and follow-up assessments are shown in Fig. 3. After seven training sessions, adherence measured with the VERITAS-Pro showed a minimal improvement from baseline 64 points up to 62 points (*p* = 0.92). Quality of life (SF-36) showed clinically relevant improvements on five of the nine domains. Two mental domains showed a large improvement: role-emotional (83 points), role-physical (63 points) and social functioning (13 points) due to emotional problems and social functioning related to work, daily activities and social relations. But surprisingly, domains that are considered related to ‘physical’ health such as general health (10 points) and pain (5.5 points) improved too. Furthermore, illness perception (BIPQ) improved from baseline 47 points to 39 points (*P* = 0.46) indicating a clinically relevant improvement in illness acceptance, without reaching statistical significance in this small sample.

#### Patient evaluation

All participants (*N* = 8) described that they were very satisfied and valued the training highly. All participants considered all sessions valuable, some preferred even more sessions. Participants felt more accepting towards haemophilia and felt less frustration regarding haemophilia-related limitations. They recognized themselves in the stories from others. After the training, most participants reported experiencing more joy in daily life activities and felt more comfortable to ask for help. They also mentioned that their partners experienced positive changes.

### Online training focussed on self-management: ‘Challenging your haemophilia’

#### Recruitment

Figure 2 shows the CONSORT flow diagram, the right side concerns the online training. Thirty patients were invited to participate in the training. Sixteen patients asked for more information about the training. Six were enthusiastic and willing to participate (enrolment 6/30), and only three patients returned the signed informed consent (including the baseline questionnaire) (recruitment 3/30). None of the participant completed the online training (retention 0/3).

#### Feasibility

Eventually, two participants started the training. The third one never started after signing the informed consent and completing the questionnaires, and provided no explanation. The two participants who started the training only completed the first module and quit because of personal life-changing issues, instead of lack of interest. Because of this, the retention for this intervention was 0% (Fig. 2). Because of the early termination of participants, follow-up data collection was 50%. The study team evaluated the training as not feasible and therefore the training was discontinued.

#### Patient characteristics

These three participants (*N* = 3) had severe haemophilia A and had a median age of 24 years. Table 3 shows patient characteristics and baseline assessments.

#### Baseline assessments

Participants only completed the baseline questionnaire. One completed only 10/40 questions of the HEIQ. Before starting, adherence (normalized VERITAS-Pro score) median score was 56 points indicating moderate adherence. Quality of life (SF-36) domain scores varied between 62 and 100 points indicating average quality of life. Self-management (HEIQ) varied between 3.0 and 3.8 indicating moderate self-management. No follow-up data are available because of early discontinuation.

#### Patient and peer trainer evaluation

Two participants were still prepared to evaluate the training. Both were positive about the modules they completed. In their opinion, the format was easy to use, had a nice layout and design and included relevant information. Both were surprised about the lack of interest of other potential participants and the fact that the training was discontinued. One suggested to use this training for a younger population because of the bit scholastic format. The peer trainers (*N* = 3) were also contacted to evaluate the training. In retrospect, they suggested that the format was a bit too scholastic for adult participants. All peer trainers wondered if participants preferred face-to-face peer contact instead of an online training. He suggested that participants with haemophilia may not be aware about their own gap in knowledge, because they have been living with this disease their whole life. All trainers were disappointed, yet understood the early termination of the intervention.

## Discussion

This study aimed to test the feasibility and primary effect of two interventions to improve adherence to prophylactic replacement therapy in haemophilia. The interventions consisted of a (1) face-to-face training focussed on acceptance and an (2) online programme focussed on self-management. The face-to-face training was evaluated as feasible; the preliminary results were promising as adherence, quality of life and illness perception all improved. Both participants and the trainers evaluated the training very positively. The online training was evaluated as not feasible because of difficulties with the enrolment, recruitment and retention. A possible explanation could be a lack of perceived need to improve self-management in persons with haemophilia or disease-specific aspects. This result was unexpected as compared to rheumatic arthritis, where the online intervention was very successful.

Both interventions were based on the systematic and extensive research, based on the van Meijel model [31] and MRC framework [24] and extensive patient participation. This project was started with a literature review [25], followed by a problem analysis [4] and a need analysis [12]. Consequently, both interventions were based on evidence-based interventions with established effectiveness [16, 23, 32–34]. The involvement of the Netherlands Haemophilia Patient Society (NVHP) included three sessions with patient panels during the development phase, testing of the interface of the online training and participation as trainers in the online intervention. The trainers for both interventions (face-to-face and online) received specific training during the development phase. Another strength of this study lies in the use of two different methods for adherence assessment. The literature recommends the use of different approaches for evaluating adherence in patients with chronic conditions (Lam & Fresco [35]). The VERITAS-Pro [26] is the only disease-specific adherence questionnaire available currently. To provide more detailed information, we additionally assessed adherence according to the Delphi definition [3]. For the main study, evaluation of pharmacy records will be added. The study is limited by the small target population; severe haemophilia is a rare disease (1:5000 males), but non-adherence is widely prevalent at 57% [4, 36]. An additional important aspect is that the face-to-face training is conditional on the willingness of these men to work on their personal problems.

Concerning the face-to-face group training, this is the first ACT-based training in haemophilia. However, there is extensive experience with ACT in other chronic conditions. A systematic review showed that ACT is effective in several chronic conditions (e.g. depression, OCD, multiple sclerosis, diabetes) [32, 33], with a large effect sizes, even increasing up to 6 to 12 months after the intervention [33]. This current feasibility study had just one follow-up measurement 2 months after the last session (i.e. 6 months after the first session), and the main study will assess participants at 6 and 12 months after the training. Graham et al. stated that ACT has promising effects on self-management, but reported that the effect of ACT on adherence is insufficiently demonstrated [34]. Moreover, these authors recommended the use of a randomized controlled trial (RCT) (ACT versus another behaviour strategy) instead of other study designs [34]. The preliminary results of this study showed hardly any effect on adherence (mean difference 2 points), yet the major change was on quality of life. The RCT design was considered, yet in our opinion it was unethical to let participants with problems with acceptance ‘wait’ in a control group. An RCT comparing two interventions was impossible as no other intervention to improve illness acceptance in haemophilia was available. A recent Cochrane review evaluated several psychological interventions for people with haemophilia [37]. It included all psychological interventions, targeted at individuals, in groups, or families [37]. This review only reported on self-administered interventions provided by technologies like a DVD and computer.

Why did the online training fail? Several potential reasons for failure were identified: age, gender and disease-specific differences. The online training was based on the comparable training for rheumatic arthritis (RA) [22, 32]. The training for RA was evaluated in both a feasibility study and a RCT [32, 38]. The feasibility study showed that the training was suitable for young adults, without recruitment problems (52% response rate). This is also in line with a recent Cochrane review which reported that online psychological interventions are effective in young people but more difficult in adults [37]. At the time our intervention was designed, the RCT in RA was ongoing and we were unaware of its results. The eventual RCT turned out not effective related to self-efficacy but was evaluated appreciated and valuable for the participants. They included mostly young females (88%, mean age of 19 years). The Dutch RA population is nine times larger and has a larger incidence and prevalence than the Dutch haemophilia population [39]. Some studies have reported that men prefer less information about their disease, in comparison to women, and that men report lower disease-specific health literacy [40–43]. Another aspect affecting recruitment may be time since diagnosis: in diabetes, newly diagnosed (< 1 year) patients were more willing to participate in self-management interventions than those diagnosed 2–3 years ago [44]. Based on our experiences, it is recommended to create more awareness in disease and gender differences related to selecting an intervention.

This study has clinical and research implications. In our opinion, the results of the present study provide sufficient reasons (preliminary results, feasibility and evaluation) for continuation of the face-to-face group training into a national pre-post-test study. To evaluate the long-term effects of this intervention, data collection will be extended to 12 months follow-up in the main study. If the face-to-face group training is effective, it could be applied in daily healthcare for both severe and non-severe haemophilia patients struggling with acceptance. For clinical practice, the positive feedback from both patients and their relatives concerning the face-to-face group training has inspired us to continue. We now believe that this kind of acceptance intervention could improve daily healthcare. This acceptance and commitment therapy training is not a country- and language-specific training [3, 4]. In our opinion, this intervention can be used in other (European) countries after proper translation. The online training was evaluated unfeasible in its current format. Based on participant evaluation, we adapted it for young teenagers who are learning self-infusion. Instead of printed information used in the past, children and parents can access an online programme with four modules. It includes educative videos, written information and tests. Nurses are able to review patients’ status and answer questions online.

## Conclusion

This paper described the feasibility of two tailored interventions concerning acceptance (face-to-face) and self-management (online) in people with severe haemophilia. Both interventions were adapted from already existing and effective interventions. The face-to-face training was evaluated as feasible with promising preliminary results (especially on quality of life). The online training was evaluated as not feasible because of recruitment problems, and therefore, it was stopped. The failure of the online training may be attributed to age, gender and disease-specific differences between the already effective intervention and this tailored intervention in patients with severe haemophilia.

## Data Availability

The datasets used and analysed during the current study are available from the corresponding author on reasonable request.
